# How to grade child health in Brazil? Comparison of the results from the Global Burden of Diseases 2015 in Latin America. A call for papers: Como classificar a saúde da criança no Brasil? Comparação dos resultados do Global Burden of Diseases 2015 na América Latina. Convocação de estudos

**DOI:** 10.1590/1516-3180.2017.1352010217

**Published:** 2017-04-20

**Authors:** Paulo Andrade Lotufo

**Affiliations:** I MD, DrPH. Full Professor, Department of Internal Medicine, Faculdade de Medicina da Universidade de São Paulo (FMUSP), São Paulo (SP), Brazil.

Several papers comparing health indicators around the world, derived from the Global Burden of Diseases (GBD) study 2015, were published in October 2016. One of these, authored by the GBD 2015 Child Mortality Collaborators, addressed global, regional, national and selected subnational levels of stillbirths, neonatal, infant and under-five mortality, 1980-2015.^1^ This article made it possible to compare mortality rates (per 1000 live births) among Latin American countries in 2015, in terms of neonatal deaths (up to the 27^th^ day of life), postneonatal deaths (from the 28^th^ day to one year of age), deaths at the ages of one to four years and deaths at ages of under five years.^2^ Moreover, it was possible to compare the pace of change among those rates over the years, by comparing three periods: 1990-2000, 2000-2015 and 1990-2015; and thus, to ascertain which countries reached Millennium Development Goal 4 (MDG-4), i.e. an annualized rate of decrease in the under-five mortality rate of 4.4%. Particularly, our focus relates to Brazil’s performance during the period 1990-2015, among 17 Latin American countries.

**Figure 1:**
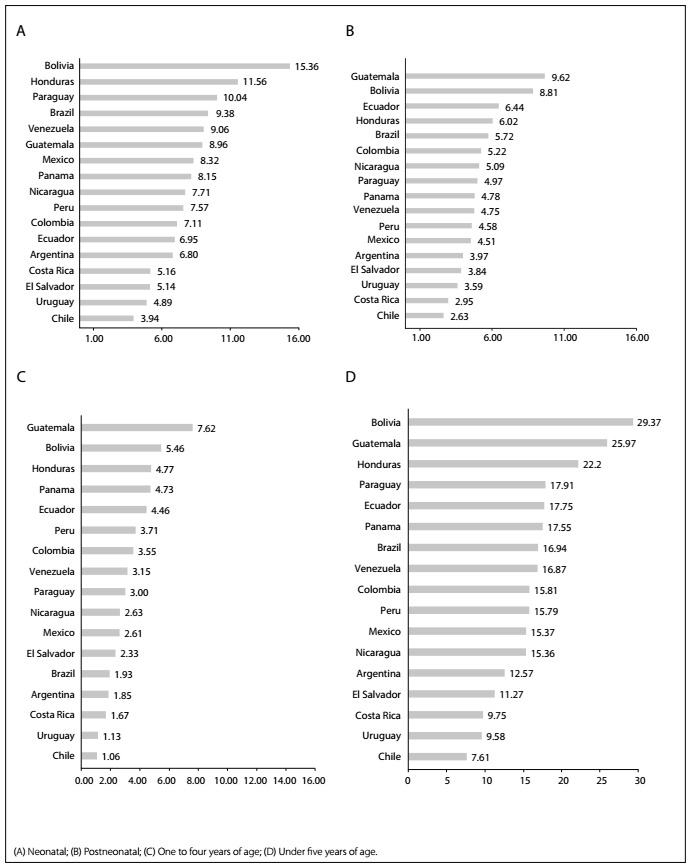
Mortality rates (per 1000 live births) in Latin American countries in 2015.

Figure 1 shows that Brazil was ranked 14^th^ for neonatal mortality (A) with rates 140% higher than Chile, which had the lowest rate. For postneonatal mortality, Brazil ranked 13^th^ with rates 120% higher than Chile, which was again the country with the lowest rate. However, the picture for the age stratum from one to four years (B) differed from the traditional infant mortality category of under one year of age, such that Brazil was in fifth place, after Chile, Uruguay, Costa Rica and Argentina.

Table 1 shows the annualized reduction in under-five mortality rates. Peru, El Salvador, Nicaragua, Bolivia, Guatemala and Brazil were the only countries with decreases of 4.4% per year from 1990 to 2015, thus reaching MDG-4. However, the pace of rate reduction was faster over the period 1990-2000 (-5.12%) than in 2000-2015 (-4.08%).

**Table 1:**
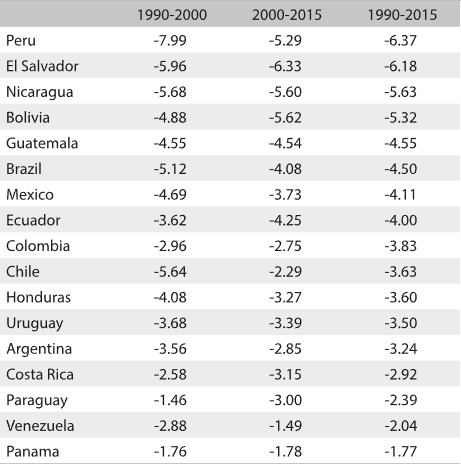
Annual percentage reduction in mortality rates among children under five years of age in Latin American countries

This same article^2^ enabled comparisons of prespecified under-five mortality rates among Latin American countries and within Brazil, subdivided according to its states (data not shown). None of these countries were classified in the lowest category of under-five mortality rates (i.e. fewer than five deaths per 1000 live births), as observed in Canada, the United States, Western Europe, Australia, Japan and South Korea. Only Chile and Uruguay were ranked in the category of 5-10 deaths per 1000 live births. Almost all Latin American countries were ranked in the third stratum (10-20 deaths per 1000) including all Brazilian states located in the South, Southeast and Center-West and some in the North and Northeast. The fourth level (20-30 deaths per 1000) was presented in Bolivia, Guatemala, Honduras and the Brazilian states of Acre, Amapá, Maranhão, Piauí, Ceará, Bahia, Alagoas and Pernambuco.

From this summary, readers can grade the quality of child health in Brazil over recent years. The Journal is calling for more articles discussing “Child Health in Brazil”.
